# Elucidation of a Physiological Adjustment Effect in a Forest Environment: A Pilot Study

**DOI:** 10.3390/ijerph120404247

**Published:** 2015-04-17

**Authors:** Chorong Song, Harumi Ikei, Yoshifumi Miyazaki

**Affiliations:** Center for Environment, Health and Field Sciences, Chiba University, Kashiwa-no-ha 6-2-1, Kashiwa, Chiba 277-0882, Japan; E-Mails: crsong1028@gmail.com (C.S.); ikei0224@ffpri.affrc.go.jp (H.I.)

**Keywords:** individual difference, initial value, walking, blood pressure, pulse rate, urban environment

## Abstract

There is a significant positive relationship between exposure to forest environments and physical and mental health. However, there are individual differences within these responses, and this phenomenon has posed questions in various fields. Here, we show that the physiological effect of a forest environment can differ depending on a subject’s initial values and that forests have a physiological adjustment effect close to an appropriate level. Subjects with high initial blood pressure and pulse rate showed a decrease in these values after walking in a forested area, whereas those with low initial values showed an increase. There was no physiological adjustment effect observed in an urban area; thus, these effects are specific to a forest environment.

## 1. Introduction

At present, most people live in urban areas, and they are expected to do so in the foreseeable future [1]. From an evolutionary perspective, urbanization is a very drastic change that has occurred over a very short period of time. Rapid urbanization and artificialization have caused environmental changes such as increase in traffic, air and water pollution, exhaustion of local resources, and decreased agricultural land and natural open space [2]. These environmental changes are a threat to human health and quality of life [2,3]. Furthermore, the rapid spread of information technology over recent years has caused an increase in stress, referred to by Brod as “techno stress” [4]. Many mental disorders and cardiovascular diseases are closely related to stress, and many studies have shown a negative physiological impact of stress on humans [5,6,7].

In recent years, the primary focus of healthcare has shifted from treatment of diseases to health promotion, disease prevention, and improved quality of life. Previous studies have focused on the role of nature in promoting human health and well-being. Through centuries of evolution within the natural environment, humans have adapted to their natural surroundings [8,9]. This tendency of humans to be close to nature implies that contact with nature may be an important component of well-being [10].

Many studies have demonstrated significant positive psychological and physiological benefits of interaction with nature. It has been shown to help in recovery from attentional fatigue and stress to improve the emotional state [8,11]. Studies of the physiological effects of relaxation in forest environments have demonstrated that staying in forests can decrease cerebral blood flow in the prefrontal cortex [12], reduce blood pressure [13] and pulse rate [13,14], increase parasympathetic nervous activity [14,15], suppress sympathetic nervous activity [14,15], decrease salivary cortisol concentrations of stress hormone [13,14], and enhance natural killer cell activity, thereby improving immune functions [16]. However, individual differences within these effects have been noted [17], and this phenomenon has posed several questions in various fields, which must be clarified. In our study, we focus on the differences in individual responses to a forest environment and an urban environment by using the law of initial value [18,19], which is the extent and direction of response of a physiological function relative to initial measures.

## 2. Methods

**Figure 1:**
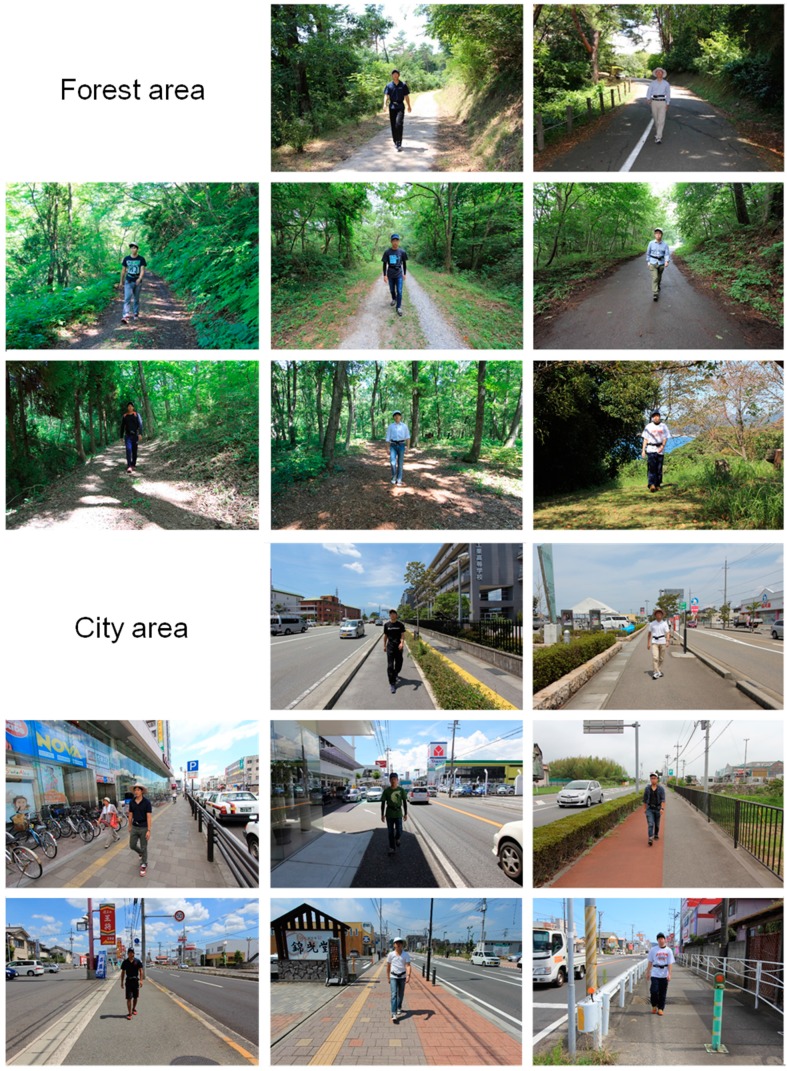
Experimental scene in a forested and city area.

We performed “within-subject” experiments. The experiments were conducted in forest and city areas in eight locations in Japan. Safe, developed forest areas were selected in each region and city areas were downtown or near a Japan Railway (JR) station. Figure 1 shows an experimental scene in a forested and city area.

Ninety-two male Japanese university students (aged 21.5 ± 1.7 years; Table 1) participated in this study. This study was performed according to the regulations of the Ethics Committee of the Center for Environment, Health, and Field Sciences, Chiba University, Japan. The subjects were fully informed about the aims and procedures involved before the experiment, and all signed an agreement to participate in the study. Alcohol and tobacco were prohibited on the day of experiment.

Systolic blood pressure, diastolic blood pressure, and pulse rate were measured by an oscillometric method using a digital blood pressure monitor (HEM1010; Omron, Japan). We measured each physiological index three times; mean values were used for comparative purposes.

Twelve subjects were deployed in each experimental region on the morning of the experiment. Subjects were randomly divided into two groups of six subjects. On the first day, one group performed the experiment in the forest area, and the other performed the same experiment in the urban area. On the second day, participants switched field sites to eliminate order effects. On arrival, subjects would await their turn in a waiting room and eventually be taken, one by one, to the experimental site. After having rested in a chair for 5 min, each subject’s blood pressure and pulse rate were assessed as the “before-walking” value. They then took a walk along the given course in the forest or urban area for 15 min. After walking, the participants rested for 5 min in a chair, after which the blood pressure and pulse rate were measured as “after-walking” value. There were no significant differences between average walking speeds in different environments.

We analyzed the correlation between the initial indicator measurements and changes observed in them following the forest/urban walking experiences. The Pearson correlation test was used to analyze the correlation between the initial values (before walking in a forest/urban area) and the changes (after walking in a forest/urban area − before walking in a forest/urban area). Statistical analysis was performed using SPSS 20.0 (IBM Corporation, Armonk, NY, USA). In all cases, *p* values < 0.05 were considered statistically significant.

**Table 1:** Subject information.

Parameter	Value (Mean ± SD)
Total sample number	92
Sex	Male
Age (years)	21.5 ± 1.7
Weight (kg)	66.6 ± 9.5
Height (cm)	173.9 ± 6.0
BMI (kg/m^2^)	22.0 ± 2.7

## 3. Results

**Figure 2:**
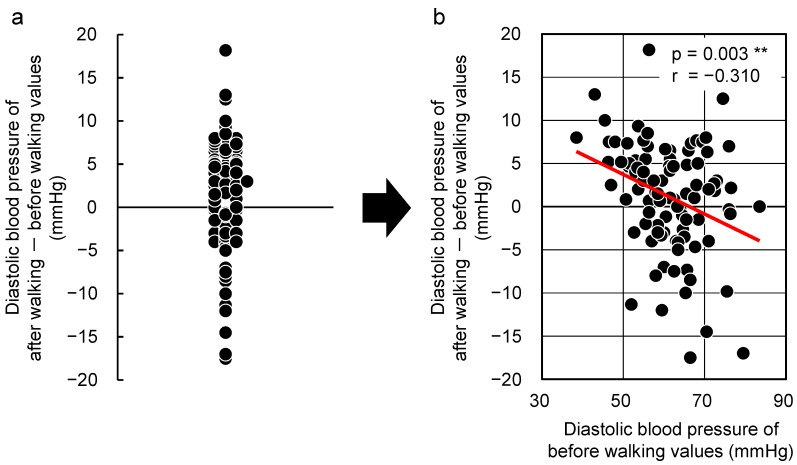
Changes observed with respect walking in a forested area. Individual differences (**a**) and the relationship between the “initial value” and the “changes after walking in a forested area” (**b**) with respect to diastolic blood pressure (n = 92). ** *p* < 0.01 by Pearson correlation test.

Figure 2 shows the large extent of individual differences in diastolic blood pressure after walking in a forest environment (a) and the relationship between the initial values and changes in diastolic blood pressure after walking in a forest environment (b). There was a negative correlation between the initial values and the changes observed in them after walking in the forest environment (r = −0.310, *p* = 0.003). Subjects whose initial diastolic blood pressure was high showed a decrease in this value after walking in a forest environment, whereas those whose initial diastolic blood pressure was low showed an increase in the value.

**Figure 3:**
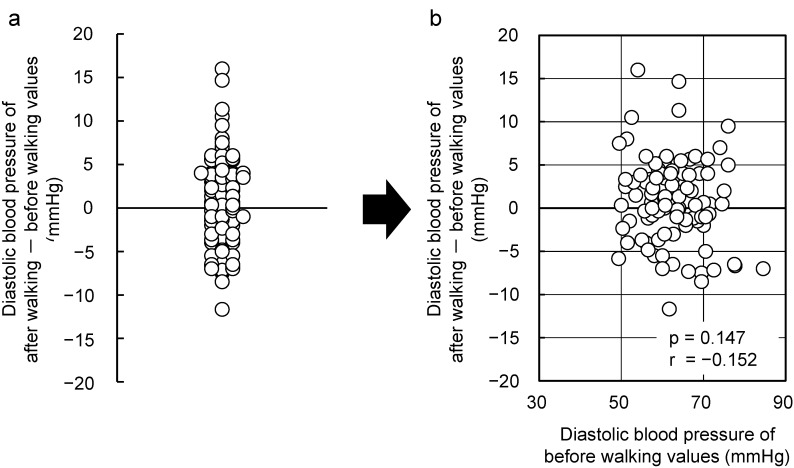
Changes observed with respect walking in an urban area. Individual differences (**a**) and the relationships between the “initial value” and the “changes after walking in an urban area” (**b**) with respect to diastolic blood pressure (n = 92). Pearson correlation test.

Figure 3 depicts the results after the subjects walked in an urban environment. Large individual differences are observed. However, no correlation could be established between the initial values and changes after walking in an urban environment (r = −0.147, *p* = 0.152).

In systolic blood pressure results, there were negative correlations between the initial values and the changes after walking both two environments (forest environment: r = −0.396, *p* = 0.000; urban environment: r = −0.264, *p* = 0.011).

**Figure 4:**
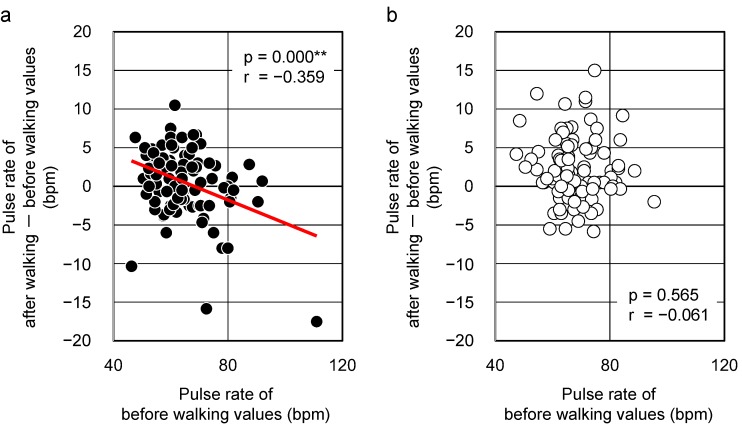
The relationship between the “initial value” and the “changes after walking” in a forested area (**a**) and urban area (**b**) with respect to pulse rate (n = 92). ** *p* < 0.01 by Pearson correlation test.

The pulse rate was also measured. There were negative correlations between the initial values and changes observed in the pulse rate after walking in a forest environment (r = −0.359, *p* = 0.000, Figure 4a). Subjects whose initial pulse rate was high showed a decrease in this value after walking in a forest environment, whereas those whose initial pulse rate was low showed an increase in this value. However, no correlation was established between the initial values and the changes after walking in an urban environment (r = −0.061, *p* = 0.565, Figure 4b).

## 4. Discussion

Our data revealed that the physiological response can differ depending on the initial values of a subject’s measured parameters and that the forest environment has a physiological adjustment effect that is close to the appropriate level.

The “law of the initial value” was proposed by Wilder in 1931 and used in many subsequent studies [18,19]. Lacey [20] examined the relationship of the initial values and changes in blood pressure and heart rate caused by a stressor and reported that subjects whose initial values were high responded poorly to function-raising stimuli such as a stressor. Hord *et al.* [21] demonstrated the correlations between the initial values and changes in heart rate and respiratory rate caused by a stressor. They found that there were significant correlations between the initial values and the changes.

On the other hand, most studies have focused on the relationship between initial measures and responses to function-raising stimuli [20,21,22,23,24]. Few studies have focused on the relationship between the initial value and the response to function-depressing stimuli, such as forest therapy. Forest therapy is a health promotion method that uses medically proven effects of forests, such as relaxation, which can improve physiological health. Tsunetsugu and Miyazaki [25] reported a significant negative correlation between a subject’s initial salivary cortisol concentrations and the extent of changes when walking in a forest environment. Lee *et al.* [26] demonstrated a significant negative correlation between a subject’s initial salivary immunoglobulin A concentrations and the relative changes when walking in or viewing a forest environment; subjects with higher initial concentrations showed a reduction in concentrations, whereas those with lower initial concentrations showed only smaller decreases and some increases.

As the modern human environment encourages busier and, hence, more stressful lives, therapeutic approaches, such as function-depressing stimuli, are considered to be very important. Our findings indicate that walking in a forest environment triggers a physiological adjustment effect that is close to an appropriate level. The subjects whose initial blood pressures and pulse rates were high showed a decrease in these values after walking in a forest environment, whereas those whose initial values were low showed an increase. These results agree with those of previous studies on the relationship between initial values and the changes in response to function-depressing stimuli [25,26]. Furthermore, this study clarified that there was no physiological adjustment effect in the urban environment; thus, these effects are specific to the forest situation. We identified physiological adjustment effects in a forest environment for the first time. Our results also will contributes to the promotion and maintenance of human health and well-being.

On the other hand, this study has limitations. The reasons why those subjects with initially high blood pressure and pulse rates showed a decrease in these values after walking in a forest environment, whereas those whose initial values were low showed an increase, and why there was no physiological adjustment effect in the urban environment, have not been identified. However, we suspect that these results are related to the fact that humans have spent most time of their evolutionary history in the natural environment [9]; the human body is made for nature [9]. However, modern humans experience stress because physiological functions that are adapted to the natural environment are unable to adapt to rapid environmental changes. Therefore, by contact with nature (e.g., forests), stress is decreased. However, these changes are not detected in an urban environment, and stress continues unabated. We hope to elucidate the mechanism in the future by using a large sample size.

The present study also aimed to elucidate individual differences on the basis of the law of the initial value using the results of 92 subjects in eight locations. These findings will contribute to a better understanding of how variations in data observed in various research fields can be caused by differences in individual subjects. Furthermore, in forest therapy, consideration of individual differences is considered to be useful in designing forest therapy programs. However, to generalize these findings, further evidence-based studies using a large sample, including various subject groups, are required. Furthermore, the statistical correlation in the present study is not so high. Therefore, further study on this topic with a larger study sample is necessary.

## 5. Conclusions

This study investigated the physiological adjustment effect in forest environment. Subjects with high initial blood pressure and pulse rate showed a decrease in these values after walking in a forested area, whereas those with low initial values showed an increase. Forest walking has physiological adjustment effects, close to an appropriate level. However, there was no physiological adjustment effect observed in an urban area; thus, these effects are specific to a forest environment.
